# Mindfulness – The Missing Link in the Relationship Between Leader–Follower Strategic Optimism (Mis)match and Work Engagement

**DOI:** 10.3389/fpsyg.2018.02444

**Published:** 2018-12-04

**Authors:** Aldijana Bunjak, Matej Černe

**Affiliations:** ^1^School of Management, University of St. Gallen, St. Gallen, Switzerland; ^2^Faculty of Economics, University of Ljubljana, Ljubljana, Slovenia

**Keywords:** strategic optimism, leader–follower (mis)match, implicit leadership theory, followership, mindfulness, work engagement

## Abstract

Assuming a followership perspective and building on implicit leadership theory, this study examines the mediating role of followers’ mindfulness in the relationship between leader–follower strategic optimism (mis)match and work engagement. Specifically, we propose that a discrepancy between the respective levels of leaders’ and followers’ strategic optimism correlates with low levels of mindfulness and work engagement. A field study of 291 working professionals, using polynomial regression and response surface analysis, supports the (mis)match hypotheses. The results demonstrate that followers’ mindfulness mediates the relationship between leaders’ and followers’ matching levels of strategic optimism (whether at high-high and low-low leader-follower strategic optimism conditions) and work engagement. These findings have important implications for training and the extent to which interventions based on personal resources, such as strategic optimism and therefore mindfulness, foster higher work engagement.

## Introduction

Although leaders’ personal characteristics determine a broad range of followers’ performance outcomes (Ilies et al., 2005; Tims et al., 2011; Dinh and Lord, 2012), comparatively little is known about followership (Bligh, 2011; Uhl-Bien et al., 2014; Leroy et al., 2015) and how followers conceive their cognitive attributes relative to leaders’ characteristics. Followers are matched with a leader possessing either similar or strikingly different cognitive characteristics, and such a dyadic situation at work influences the joint relationship dynamics, thereby impacting followers’ work outcomes, i.e., work engagement (Shamir, 2007; Schyns and Sanders, 2007; Gooty et al., 2009; Felfe and Schyns, 2010).

In the last few years, positive organizational scholarship (e.g., Cameron and McNaughtan, 2014) and positive organizational behavior (e.g., Luthans and Avolio, 2009) have gained momentum in research. Their representing constructs, such as optimism (Youssef and Luthans, 2007; Avey et al., 2011), are receiving increasing attention and have been shown to be associated with desired work-related employee outcomes (Luthans et al., 2007; Avey et al., 2009). Specifically, strategic optimism refers to a cognitive strategy used by individuals that is characterized by the following: they enjoy a sense of control over a given situation; they can perform with minimal anxiety; and they set high expectations, on the basis of past success, thereby avoiding reflection on potentially negative outcomes (Chang et al., 2009). The question then arises whether strategically optimistic followers feel better about their leaders when they perceive them as like-minded relative to themselves. Even more important is the effect on followers’ work engagement in the opposite condition; that is, when followers’ perception of leaders’ strategic optimism does not match.

Building on previous research on implicit leadership theory (ILT; Shondrick et al., 2010; Epitropaki et al., 2013), followers compare the characteristics of their actual leader to their “ideal” conception of a leader, and any discrepancy between the two modifies (positively or negatively) the followers’ impressions of their leader (Lord and Hall, 2003; Lord and Brown, 2004). This perceived similarity is a crucial factor in establishing positive leader–follower relationships (Cornelis et al., 2006; Brouer et al., 2009), in particular because followers’ subjectively perceived similarity (i.e., individuals’ beliefs about how similar they are to a leader) is even more important than actual resemblance (Watson et al., 2000; Montoya et al., 2008; Fischer, 2009).

Following the preceding logic, we argue that followers with high or low strategic optimism are prone to be affected by their leaders’ strategic optimism. Although self-evaluation (i.e., followers’ intrapersonal evaluation) is considered to be an implicit process (Pelham et al., 2002), evaluation of others (i.e., interpersonal evaluation) triggered by welcoming information of perceived similarity is instead a controlled process which involves conscious effort and awareness (Von Hippel and Trivers, 2011). Specifically, we argue that the perceived similarity in cognitive strategies (Harrison et al., 2002) triggers corresponding psychological processes, such as enhanced attention and awareness, which is further regarded as the fundamental component of mindfulness (Teasdale et al., 2002; Brown and Ryan, 2003; Kabat-Zinn, 2003; Baer et al., 2006; Grabovac et al., 2011; Mikulas, 2011). Positive association with self (Pelham et al., 2005) may stimulate followers to pay more profound attention, including higher levels of awareness of personal attraction to a leader (Kristof-Brown et al., 2005; Edwards and Cable, 2009). Such interplay is particularly relevant to work-related outcomes, such as work engagement (Leroy et al., 2013; Malinowski and Lim, 2015). This is so because followers who identify more closely with a leader are more responsive and willing to subordinate themselves to that leader (Lord and Maher, 1991; Eagly and Karau, 2002; Lord and Hall, 2003; Brouer et al., 2009).

The intended contributions of this study are three-fold. First, by investigating the proposed expectation-perception model with the focus on followers’ subjectively perceived similarity with the leader (Toma et al., 2012), we contribute to the literature related to ILT and followership by explaining when and why a (mis)match between followers’ and leaders’ strategic optimism is likely to occur (Schyns and Meindl, 2005; Schyns and Day, 2010), and how this mechanism supports followers’ awareness of similarities by fostering the joint relationship and, in turn, followers’ work engagement. Second, our study contributes to the research on cognitive strategies (i.e., strategic optimism) by proposing that the positive effect of a follower’s strategic optimism depends on a leader sharing a similar level of strategic optimism, thereby contributing to the follower’s awareness of the suitability of the leader–follower relationship and heightened awareness. Third, we contribute to the mindfulness literature by providing insights into the conditions under which mindfulness may emerge, flourish and lead to beneficial outcomes. Specifically, assuming the follower’s perspective, positive association between oneself and the leader spurs emergence of awareness (i.e., heightened mindfulness). Therefore, the main message of this study is that a leader–follower strategic optimism match, through mindfulness, should foster superior levels of employee work engagement.

## Theory and Hypotheses

Studies have criticized traditional leadership theories for placing undue emphasis on the impact of leaders’ characteristics on followers’ attitudes and behaviors (Graen and Uhl-Bien, 1995; Uhl-Bien et al., 2014). As a response, followership literature is concerned with articulating effective follower characteristics, follower behaviors, and outcomes relative to leaders (Meuser et al., 2016). Followership theory argues that leadership cannot be fully understood without a meaningful consideration of the followers’ impact on the leadership process (Dvir and Shamir, 2003; Sy, 2010).

Even though leaders and followers influence each other’s perceptions (Pirola-Merlo et al., 2002), research has shown that followers’ self-evaluation significantly influences their assessment of the leader (Hall and Lord, 1995; Howell and Shamir, 2005). The way that one views oneself (i.e., the psychological process of positive association with self) significantly determines the way one perceives others, i.e., how a follower sees a leader (Keller, 2003). Naturally, leaders are not all perceived as leaders, and this leadership perception depends enormously on the leader’s actual characteristics, behavior, and skills (Cavazotte et al., 2012) and on followers’ conceptions of an ideal leader (Epitropaki and Martin, 2004). A leader is a product of the overlap between followers’ identification with their ideal conception of a leader and the leader’s actual characteristics (Lord et al., 1984). This ideal image of a leader is formed through previous interactions with different leaders (Ritter and Lord, 2007) and role models, such as parents (Keller, 2003).

Dinh and Lord (2012) observed that followers use implicit leadership principles to arrive at conclusions about others’ leadership based on their personal characteristics. In other words, followers’ perceptions of leaders’ characteristics are crucial to their categorization of a leader (Shondrick et al., 2010). Implicit leadership is a process whereby perceivers, based on cognitive structures, subjectively observe the world, including the characteristics of others, around them (Epitropaki et al., 2013). The process enables the observation of a leader’s actual characteristics, as well as their “ideal” characteristics, to make sense of the leader’s behavior (Medvedeff et al., 2007). A match between leaders’ characteristics and the characteristics of followers’ leader prototypes (Lord et al., 2001) will prompt followers to accept the leader as someone truly capable of leading them and their team. The match will promote followers’ positive behavior and foster good attitudes toward their leader. By contrast, a mismatch will result in negative work-related outcomes, such as high turnover and followers’ general dissatisfaction (Engle and Lord, 1997).

Furthermore, research shows that individuals prefer to socialize with individuals who share common behavior, preferences, and personality dimensions, such as strategic optimism (Reis et al., 2000; Shondrick et al., 2010). Additionally, van Quaquebeke et al. (2011) found that when followers are asked to evaluate their leaders, the evaluation is not only influenced by self-perception, but also by the extent to which followers perceive themselves as similar to their leaders. Thus, followers who regard their relationship with a leader as poor and have (mis)matched expectations may experience reduced commitment to the leader and the organization, thereby resulting in negative work outcomes, i.e., decreased levels of work engagement (Van Breukelen et al., 2002).

### The Congruent Effect of Leader–Follower Strategic Optimism on Followers’ Mindfulness and Work Engagement

Unlike the trait-like form of optimism, which is relatively stable and rigid (Scheier and Carver, 1987), state-like strategic optimism is a cognitive strategy associated with a specific problem or goal within a particular situation and temporal context (Norem, 2001). Research has proven strategic optimism to be a powerful motivator, suggesting that this cognitive strategy enables individuals to set high expectations, and avoid reflecting unnecessarily on upcoming events (Norem and Illingworth, 1993). This coping mechanism is even more relevant nowadays, when leaders and followers have virtually no choice but to operate under great stress and anxiety in different work situations (Blaskovics, 2014). Similarly, a leader–follower match in strategic optimism is equally important, because a match in leader–follower cognitive strategy seems to represent a precondition for a healthy relationship between the two component parties in this association (Bunjak and Černe, 2018).

If we peer through the lens of ILT, expectations set by a strategic optimist (Spencer and Norem, 1996) will greatly depend on expectations of a leader (Biddle, 1979). Consequently, when leaders and followers share common expectations that are shaped by cognitive characteristics, such as strategic optimism, followers will find their jobs more pleasurable than they will in a mismatch situation. Leaders who score low in strategic optimism with followers who maintain high strategic optimism will be regarded as unpleasant, overly nervous, negative, and controlling (Rowold and Schlotz, 2009). Similarly, a leader who scores high in strategic optimism might be regarded as insufficiently consistent in and serious about their work (Taylor and Brown, 1988).

In the same vein, leader–member exchange (LMX) research builds on implicit theory, noting that individuals will thrive most at work when they perceive the leading party to contribute equally or more to the relationship (Buunk et al., 1993). Accordingly, with more consistency, and thus more similarity between the leader prototype (i.e., followers’ implicit expectations) and the actual leader’s characteristics (Scott and Brown, 2006), the more the leader is regarded as contributing to the LMX relationship (van Gils et al., 2010). Moreover, followers will more easily relate to and understand information about the leader when the specific leader’s characteristics are similar to (i.e., matched with) the followers’ implicit leadership expectations (Shondrick et al., 2010). For example, if followers perceive themselves as being strategic optimists, they will expect their leader to act similarly (Montoya and Horton, 2013), to provide a corresponding contribution to the joint relationship, thereby maximizing the work outcome (i.e., follower’ work engagement).

Similarly, ILT enables individuals to make sense of leaders’ characteristics (Shondrick et al., 2010) based on self-perception relative to others (Junker and van Dick, 2014) and previous social experiences (Keller, 1999). Hence, based on ILT, a match of leader–follower strategic optimism implies high levels of self-perception and self-perception relative to others, that is, perspective-taking (Galinsky and Moskowitz, 2000; Parker and Axtell, 2001). More specifically, perspective-taking refers to an individual’s ability to see the world as others see the individual (Edwards et al., 2017).

We therefore believe that an ILT explained in terms of coordination between “I” and “You” [i.e., as with a (mis)match between leader and follower strategic optimism] also involves features of mindfulness, that is, enhanced attention to and awareness of self (i.e., follower) relative to other (i.e., leader). Moreover, in our study, mindfulness regarded as a state [rather than as a trait, agreeing with a recent meta-analysis that identified the malleability of this construct (Eberth and Sedlmeier, 2012)], emerges only when attention to present circumstances (in this sense, attention of leaders’ characteristics) is intentionally evoked (Chiesa, 2013). In this state of mind, an individual takes no action, but simply acknowledges and observes the changing flow of thoughts as they arise moment by moment (Kabat-Zinn, 2003). Specifically, the very moment of followers’ perceived similarity with the leader [in our study, this was intentionally evoked by asking participants to evaluate their leaders’ deep level psychological characteristics (Harrison et al., 2002)] such as strategic optimism, triggers attention to and awareness of present reality [i.e., (mis)matched leader–follower cognitive characteristics], and thus mindfulness (Brown and Ryan, 2003). This leads to our hypotheses, formulated as follows:

Hypothesis 1a: When leader and follower match in strategic optimism at low levels, the level of follower’s mindfulness is high.
Hypothesis 1b: When leader and follower match in strategic optimism at high levels, the level of follower’s mindfulness is high.

**FIGURE 1:**
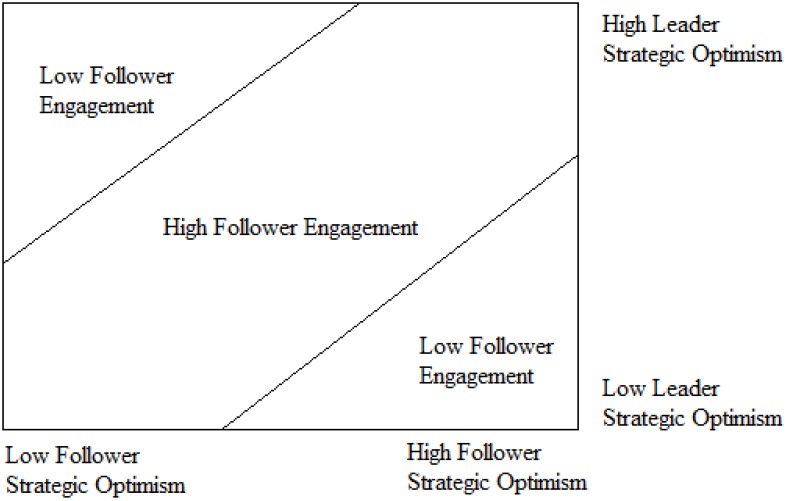
Follower engagement (mis)match model.

Studies have shown that leadership is in the eye of beholder (Gooty et al., 2009). The greater the consistency of the components of follower perception, the more efficiently leaders are at directing follower attitude and work-related outcomes (Fleenor et al., 2010). Matching at deep-level similarities, such as leader–follower strategic optimism (Oakes et al., 1991), invokes mindful information processing, which lends clarity to the interpretation of explicit leader behavior (Lord and Maher, 1991). Moreover, followers who acknowledge similarities with leaders’ characteristics and relationship fit will experience commitment and engagement (Chalofsky and Krishna, 2009), as well as identification of the benefit of a leader as their own (Sluss and Ashforth, 2007). In addition, follower mismatch with a leader’s characteristics (see Figure 1: either follower strategic optimism > leader strategic optimism or follower strategic optimism < leader strategic optimism) will result in follower turnover, absenteeism, lack of commitment, and poor engagement (Jones and Harter, 2005; Albrecht and Andreetta, 2011).

Particularly, matched expectations (i.e., similarity with the leader) evoke awareness of such inner experience, making people receptive and attentive to present occurrences (Brown and Ryan, 2003). After all, followers’ will prioritize and be open-minded about welcome (i.e., matched strategic optimism) more than unwelcome (i.e., mismatched strategic optimism) information about their leaders (Von Hippel and Trivers, 2011). On the other hand, mindfulness channels awareness of thoughts, sensations, and individuals’ attention to the present experience (Bishop et al., 2004; Feldman et al., 2010) in an open manner, which may improve cognitive barriers of ILT, such as inconstant information (Lord and Maher, 1991), and help individuals to keep processing actual information about leaders’ characteristics and behavior at all times (Carlson, 2013; Pircher Verdorfer, 2016). Moreover, mindfulness facilitates prioritizing, limiting, and directing information (Jha et al., 2007); reduces mind wandering (Mrazek et al., 2013); and enhances cognitive memory and operationalization of information (Zeidan et al., 2010).

Finally, when a follower’s self-perception of strategic optimism, relative to a leader’s strategic optimism is strongly aligned, the individual perceives the leader as a part of their self-concept (Lord and Brown, 2001). Such individuals tend to contribute beneficially to their leaders and organization (Kristof-Brown et al., 2005), thereby increasing work engagement (Gardner et al., 2005). Likewise, engaged employees tend to be regarded as immersed, fully present, and attentive in their activities (Rich et al., 2010), and mindfulness further enhances those positive experiences, by making them clear and vivid as they occur (Brown and Ryan, 2003). In other words, alert attentiveness that describes evaluation of the leader can be fully attendant to by a followers’ mind that is aware of what takes place in the present moment (Good et al., 2016). Therefore, we propose:

H2: Mindfulness mediates the relationship between leader–follower strategic optimism match and work engagement.

**FIGURE 2:**
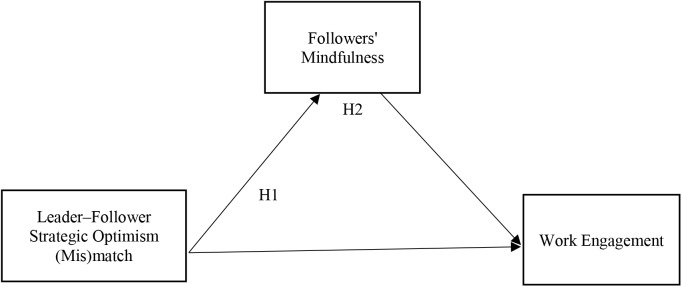
Research model with hypotheses.

Figure 2 presents our conceptual model with hypotheses.

## Materials and Methods

### Data Collection Procedure

An online questionnaire of working professionals was conducted in spring 2016. Participants were recruited via posts on social networking websites, such as Facebook and LinkedIn. Potential participants were also targeted through various groups (e.g., Happiness at Work, Business Psychology at Work, Employee Engagement, and Cognitive Neuroscience) and via personal contacts. The participants were notified that the aim of the research was to explore the dynamics perceived by employees at their workplace. After agreeing to participate, participants were directed to the survey website. The survey took approximately 10 min to complete.

### Sample

The mandatory requirement for study participation, for working professionals, was that the participants be employed. In line with our follower-centric theoretical perspective, our mode of data collection relied completely on self-reporting, from the perspective of the employees, who gave responses about themselves and their leaders. The online survey was completed by 291 employees; 65% of respondents were female, and approximately 45% were less than 35 years of age. Most of the participants had acquired a master’s level degree (44.4%), and they were from the United States (21.4%), Slovenia (19.1%), Bosnia and Herzegovina (10.6%), the United Kingdom (6.4%), and Australia (4.8%). Their main fields of employment were education (34%), finance (17.2%), the service industry (12.4%), health care (10%), and government (9.3%). The majority (55.3%) had less than 3 years of work experience with their leaders, followed by 26.1% who had 3–6 years of dyadic tenure, 9.3% with 7–10 years of dyadic tenure, and 9.3% with 11 or more years of dyadic tenure experience.

### Measures

This study used five-point Likert-type scales ranging from 1 (“*strongly disagree*”) to 5 (“*strongly agree*”). The measures were administered in the English language.

#### Follower’s Strategic Optimism

The strategic optimism scale was adapted from the defensive pessimism scale (Norem, 2001), which consists of several items that refer to the process of thinking through things, as well as items designed to measure strategic optimism. For the purpose of this study, we only selected items that measure strategic optimism. A sample item was “I go into these situations expecting the worst, even though I know I will probably do OK.” (α = 0.63).

#### Leader’s Strategic Optimism

As in the assessment of followers’ strategic optimism, the Strategic Optimism Questionnaire (Norem, 2001) was used. Because we wanted to assess how followers perceive their leaders, in the follower domain, the scale was adapted to include a referent shift to the leader. Accordingly, a sample item was “He/she goes into these situations expecting the worst, even though he/she knows he/she will probably do OK.” (α = 0.71).

#### State Mindfulness

Mindfulness was assessed using the five items with the highest factor loadings, adapted from the Mindful Attention Awareness Scale (MAAS; Brown and Ryan, 2003). Sample items included “Today, I found it difficult to stay focused on what’s happening in the present,” and “Today, I found myself doing things without paying attention.” Participants were asked to indicate the extent to which these items described their feelings and behavior during working hours (α = 0.79).

#### Work Engagement

Work engagement was assessed using the six items with the highest factor loadings adopted from the Utrecht Work Engagement Scale (UWES; Schaufeli et al., 2002), and treated as an overall index consisting of vigor, dedication, and absorption. Example items for these components were, respectively, “At my work, I feel bursting with energy,” “I am enthusiastic about my job,” and “I feel happy when I am working intensely.” (α = 0.89).

#### Control Variables

We controlled for four variables (i.e., age, gender, employee education, and tenure an employee has with their leader) in our analyses, because previous studies have shown these to be related to employee work engagement (Mauno et al., 2005; Avery et al., 2007).^1^

#### Analyses

In order to reduce potential common method bias effects, we conducted several *a priori* steps. First, our survey was part of a larger data collection, rendering respondents unlikely to guess the purpose of the study. Second, several items were reverse-coded. However, given the cross-sectional and single-source nature of our research, we also applied the marker variable test developed by Lindell and Whitney (2001) using a theoretically unrelated variable (i.e. marker variable) to adjust the correlations among the principal constructs in the model. Any high correlation of the marker variable with any other of the study’s principal constructs would indicate potential common method bias. For robustness, we separately repeated the marker variable test with an additional variable that was not included in the model [the Big 5 personality trait of conscientiousness, tapped by a short measure, presented by Gosling et al. (2003)], for which we had little or no theoretical basis to expect a relationship with the study’s principal constructs. The average correlation between the study’s principal constructs and conscientiousness (*r* = 0.09) was low and not significant, and the integration of this variable into the research model did not alter the significance of the main studied relationships, providing no evidence of common method bias.

Polynomial regression analysis and response surface modeling were applied to test the (mis)match hypotheses (Edwards and Parry, 1993). We centered the scales to reduce multicollinearity between the component measures (i.e., leader and follower strategic optimism) and their associated higher-order terms (Aiken et al., 1991). To test the mediating hypothesis, we applied the block variable approach suggested by Cable and Edwards (2004) for mediation analysis. The block variable approach involves obtaining a single coefficient that summarizes the effects of a set of conceptually related variables (Cable and Edwards, 2004). Accordingly, we constructed a block variable by first regressing the dependent variable (i.e., work engagement) on the five polynomial terms. We then used the respective weights, which were the estimated regression coefficients in the polynomial regression (i.e., *b*_1_X + *b*_2_Y + *b*_3_ X^2^ + *b*_4_XY + *b*_5_Y^2^), and combined the five terms into a block variable as a weighted composite (Cable and Edwards, 2004) that summarized the effects of leader–follower strategic optimism (mis)match on work engagement (Edwards and Cable, 2009). Lastly, we conducted mediation analysis using the PROCESS macro (Preacher and Hayes, 2004), with the block variable for strategic optimism as the independent variable, followers’ mindfulness as the mediating variable, and work engagement as the dependent variable. We examined the direct and indirect effects using bootstrap as a bias-correction percentile method with 10,000 samples (Cable and Edwards, 2004) and conducted bias-corrected confidence intervals (Edwards, 2002). The proposed mediation will be supported if the confidence interval of the indirect effect does not include zero.

## Results

Table 1 presents the descriptive statistics (means, standard deviations, and correlations) of all variables used in the study. We observed the factor structure of the focal variables, using confirmatory factor analysis procedures in AMOS software version 21. The expected four-factor solution (follower’s strategic optimism, leader’s strategic optimism, followers’ mindfulness, and work engagement) displayed a good fit with the data [chi-square (166) = 276,371, CFI = 0.951, SRMR = 0.0748, RMSEA = 0.048]^2^. The standardized factor loadings ranged from 0.34 to 0.70 for follower strategic optimism items, from 0.40 to 0.81 for leader strategic optimism items, from 0.52 to 0.83 for followers’ mindfulness, and from 0.58 to 0.92 for work engagement items.

**Table 1:** Means, standard deviations, alpha reliabilities, and correlations among variables^a,b,c^.

Variable	Mean	S.D.	Alpha	1	2	3	4	5	6	7
(1) Age	3.51	0.78	n.a.	–						
(2) Education	2.80	0.79	n.a.	0.17^∗∗^	–					
(3) Gender	1.65	0.47	n.a.	−0.02	−0.04	–				
(4) Leader–follower dyadic tenure	1.72	0.97	n.a.	0.27^∗∗^	0.04	0.07	–			
(5) Follower’s strategic optimism	3.35	0.44	0.63	−0.29^∗∗^	−0.03	0.15^∗^	−0.03	–		
(6) Leader’s strategic optimism	3.18	0.43	0.71	−0.17^∗∗^	−0.01	0.16^∗∗^	−0.01	0.31^∗∗^	–	
(7) Followers’ mindfulness	3.39	0.82	0.79	0.21^∗∗^	0.05	−0.07	−0.01	−0.26^∗∗^	−0.13^∗^	–
(8) Work engagement	3.61	0.81	0.89	0.11	0.13^∗^	0.00	0.15^∗^	−0.02	0.09	0.21^∗∗^

*^a^n = 291. ^b^Age was classified into five classes: 1 = less than 18, 2 = 18–24, 3 = 25–34, 4 = 35–54, and 5 = 55 and over. Dyadic tenure was classified into four classes: 1 = less than 3 years, 2 = 3–6 years, 3 = 7–10 years, and 4 = 11 years and over. ^c^1 = male, 2 = female ^∗∗^p < 0.01, ^∗^p < 0.05.*

### Hypotheses Testing

**FIGURE 3:**
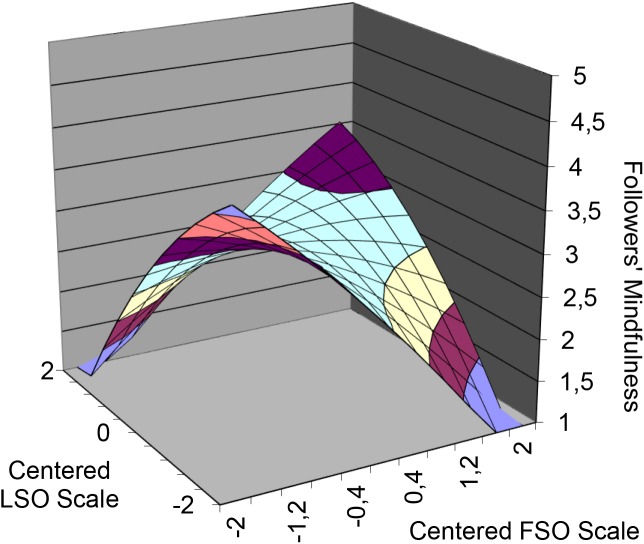
Leader–follower strategic optimism (mis)match matrix predicting followers’ mindfulness. LSO = Leader’s strategic optimism; FSO = Follower’s strategic optimism.

Hypotheses 1a and 1b predict that follower mindfulness will be higher when leader’s and followers’ strategic optimism are congruent, whether at lower or higher levels. Table 2 shows the results of the polynomial regression analysis. The curvature (Figure 3) along the line of congruence (X = Y) was positive and significant (*a*_2_ = 0.25, *p* < 0.05), whereas the curvature along the incongruence line (X = −Y) was negative, as expected, and significant (*a*_4_ = −0.59, *p* < 0.01). Therefore, Hypotheses 1a and 1b are supported.

**Table 2:** Polynomial regression analyses results predicting followers’ mindfulness.

Dependent variable	Followers’ mindfulness
Constant	2.87 (0.31)^∗∗^
Age	0.24 (0.06)^∗∗^
Gender	−0.13 (0.10)
Education	−0.00 (0.06)
Leader–follower dyadic tenure	−0.06 (0.05)
Follower’s strategic optimism	−0.08 (0.10)
Leader’s strategic optimism	−0.08 (0.09)
Follower’s strategic optimism^2^	−0.05 (0.12)
Follower’s strategic optimism × leader’s strategic optimism	0.42 (0.14)^∗∗^
Leader’s strategic optimism^2^	−0.11 (0.07)
*F*	3.29
*df*	281
*R*^2^	0.10
**Congruence (follower’s strategic optimism = leader’s strategic optimism) line**	
Slope (*a*_1_)	−0.17 (0.12)
Curvature (*a*_2_)	0.25 (0.10)^∗^
**Incongruence (follower’s strategic optimism = -leader’s strategic optimism) line**	
Slope (*a*_3_)	0.00 (0.09)
Curvature (*a*_4_)	−0.59 (0.22)^∗∗^

*N = 291. ^∗^p < 0.05; ^∗∗^p < 0.01; the items reported are standardized beta coefficients; standard errors are in parentheses.*

To test Hypothesis 2, we first computed a block variable, using the estimated coefficients predicting work engagement. We then ran a mediation analysis using the PROCESS macro (Hayes, 2013), as shown in Table 3.

**Table 3:** Results of the mediating analyses with the PROCESS macro.

Dependent variable	Followers mindfulness	Work engagement
Constant	2.88^∗∗^	2.43^∗∗^
Coefficient of the block variable (i.e., FSO, LSO, FSO^2^, FSO × LSO, and LSO^2^)	1.22 (0.38)^∗^	0.72 (0.38)^∗^
Followers’ mindfulness	—	0.18 (0.05)^∗∗^
Age	0.24 (0.06)^∗∗^	0.01 (0.06)
Gender	−0.12 (0.09)	0.00 (0.09)
Education	0.00 (0.05)	0.11 (0.05)^∗^
Leader–follower dyadic tenure	−0.06 (0.05)	0.11 (0.04)^∗^
*F*	5.49	4.89
*df*	(5, 285)	(6,284)
*R*^2^	0.08	0.09
Indirect effect of leader–follower strategic optimism (mis)match on followers’ work engagement via followers’ mindfulness	—	0.22 (0.13)^∗^ (LLCI: 0.05, ULCI: 0.57)

*N = 291. ^∗^p < 0.05; ^∗∗^p < 0.01; unstandardized coefficients are reported (standard errors in parentheses). FSO, Follower strategic optimism. LSO, Leader strategic optimism. LLCI, lower level confidence interval. ULCI, upper level confidence interval. The indirect effect was tested using bias-corrected percentile method with bootstrapping 10,000 samples. 95% confidence intervals are represented.*

Examining the mediation of follower mindfulness of the relationship between leader–follower strategic optimism (mis)match and work engagement, we generated 95% bias-corrected confidence intervals (Preacher and Hayes, 2004) for the hypothesized indirect mediating effects. The direct effect of the block variable on engagement, before the inclusion of the mediator, was significant (*b* = 1.23, *p* < 0.01). The indirect effect of the block variable of strategic optimism, on work engagement through mindfulness, was significant (*b* = 0.2248), because the confidence interval from the bootstrap analysis excluded zero [0.0457, 0.5752], supporting Hypothesis 2. Finally, the direct effect of the block variable on work engagement, after the inclusion of the mediator, was not significant (*b* = 0.73, *p* > 0.05).

## Discussion

### Theoretical Contributions

First, our study contributes to the ILT by explaining the role of cognitive characteristics (mis)match in shaping followers’ perceptions of an implicit relationship agreement (Schyns and Meindl, 2005; Shamir, 2007). The key to how the relationship will be defined depends on followers’ perceptions, and our study identified the crucial role played by a match in strategic optimism. This agrees with work by Dinh and Lord (2012), who stated that followers use ILT to reach conclusions about leadership in others, based on followers’ characteristics as well. The followers’ perceptions of leaders’ characteristics are thus very important in the process of categorizing a leader (Shondrick et al., 2010). Specifically, our study demonstrated that working with a leader who shares a similar cognitive style, while maintaining awareness of such similarities, may contribute to higher follower work engagement. Assuming a followership perspective, i.e., focusing on followers’ perceptions of themselves and of their leaders, thus represents a viable mode of applying ILT to future research.

Second, our study contributes to the research on cognitive styles by showing that the positive effect of followers’ strategic optimism depends on the extent to which followers also perceive similar levels of strategic optimism in their leader. Thus, high levels of what is a generally positive characteristic, *per se*, are not sufficient; it is necessary to provide a second view that provides a balance to that effect. When they are both in balance, in fact, at both low and high levels, our study revealed that they contribute to a follower’s awareness of the leader–follower relationship (i.e., follower’s mindfulness) and thereby to an increase in the follower’s work engagement.

Third, we contribute to the mindfulness literature by examining followers’ mindfulness as a crucial missing link in the relationship between leader–follower strategic optimism (mis)match and work engagement. Again, this notion relates back to ILT; followers’ identification with a leader and employees’ work setting is not determined solely by leader prototype (Lord et al., 1984; Shondrick et al., 2010), but also by the extent to which leaders and followers share common cognitive characteristics. Perceptions are thus crucial, and this is especially true when research is focused on the connection between mindfulness and leadership. Mindfulness represents an integral component in explaining how the match between followers’ and leaders’ cognitive characteristics leads to work engagement via followers’ awareness (i.e., mindfulness) of that match, as well as of their work setting and the cognitive style of their leaders. Mindfulness thus embodies an important phenomenon that enables individuals at work to interpret their work settings and their dyadic interactions with leaders.

### Practical Implications

The key practical implication of our study is related to ensuring leader–follower alignment in cognitive styles. Strategic optimism can be measured in advance, which should be a key informational element when establishing working follower–leader dyads or assigning followers to specific leaders. This seems to be a viable way of ensuring higher levels of employee mindfulness, and thereby promoting higher levels of work engagement, which has been shown to lead to motivation, innovation, and productivity (Schaufeli and Salanova, 2007).

The management of perceptions is also important, and represents an important aspect of the project of ensuring that the leader–follower match in cognitive styles is, in fact, interpreted as a congruence. Both participants in leader–follower dyads should be trained to prevent the development of perception biases (Černe et al., 2014; Hansbrough et al., 2015), while taking particular care to ensure that the cognitive style of a leader is made apparent to the follower through transparent and open communication. Training and development initiatives in organizations should thus focus on establishing high-quality relationships at work through communication-improving exercises and fostering the socialization process at work (Dutton and Ragins, 2017).

Mindfulness is associated with increased self-reported perspective-taking, which includes understanding the behavior of others (Dekeyser et al., 2008). Similarly, mindfulness improves communication and heightens information accuracy, thereby decreasing conflicts in relationships (Wachs and Cordova, 2007). Because followers form a leadership picture based on their judgments of the characteristics that leaders ought to have (Funder and Sneed, 1993), accurately perceived information is crucial to the establishment of functionally effective leader–follower relationships. Specifically, mindfulness training promotes the accuracy of followers’ self–other perception, which helps individuals to adjust the “self-perceived picture” of a leader in accordance with the actual one, thus evoking desirable outcomes, such as higher levels of work engagement.

It should here be noted that a follower’s optimism can be improved and cultivated, as shown by work on learned optimism (Seligman, 2011) and resource-based intervention programs (Costantini et al., 2017). Psychological capital (i.e., optimism) was able to increase positive work-related outcomes, such as work engagement. Therefore, organizational psychologists should focus on the developmental nature of optimism, as demonstrated by various positive psychology interventions and programs (Seligman et al., 2005; Costantini et al., 2017).

### Limitations and Future Research Suggestions

As is true of any study, this research is not without its limitations. In this case, the limitations relate both to the study’s empirical design and to its theoretical background, offering promising avenues for future research. First, our study was cross-sectional in nature and based on a single source (i.e., employees). Even though our research questions and constructs called for followers’ assessments of phenomena they experience at work and thereby could not be other-rated, future studies could, perhaps, aim to tap into work engagement from the leader’s perspective or could examine other individual performance measures that might be more objectively rated by other sources. Research has shown that attention can be improved by noticing novel things (Langer, 2000; Langer and Moldoveanu, 2000) or/and by low-dose mindfulness interventions, such as mindfully washing dishes (Hanley et al., 2015). However, a second and related limitation involves the causality in our proposed and tested relationships, which, although based on theory, cannot be ascertained in a sufficiently definite manner; to that end, future research should adopt longitudinal research designs (e.g., through a diary study).

Third, by focusing on leaders’ and followers’ strategic optimism, we have only scratched the surface of cognitive processing and leadership research. Additional promising constructs that could be examined, with respect to leader–follower (mis)match examination and mindfulness, include thinking styles (Cheek and Norem, 2017) and elements of positive psychological capital that depend less on cognitive processing and more on affective processing, e.g., hope, resiliency, confidence, and general optimism (Luthans et al., 2010). Future studies could also focus on conditions and outcomes in which the leader–follower mismatch, in terms of a certain characteristic, could potentially be beneficial; this would represent an even further extension of the trend in organizational psychology research of examining the boundary conditions of positive phenomena that have negative effects (Pierce and Aguinis, 2013), and vice versa.

Finally, although this model makes sense from an intuitive standpoint and provides valuable results in terms of explaining the quality of the leader–follower relationship, it is still built only on quantitatively measured followers’ perceptions. As such, qualitative data collected in future research could potentially be used to clarify some of its complex nuances. Therefore, it behooves us to continue deepening our understanding of the leader–follower (mis)match in organizations and of the outcomes and implications of this (mis)match.

## Ethics Statement

This study was carried out in accordance with the recommendations of Core Practices and Guidelines published by the Committee on Publication Ethics. The protocol was approved by the Committee of Research Ethics at the Faculty of Economics, University of Ljubljana. All subjects (field study respondents) gave written informed consent.

## Author Contributions

Both authors contributed to conceptualizing the study and its research design, collecting the data, analyzing it, and writing it up.

## Conflict of Interest Statement

The authors declare that the research was conducted in the absence of any commercial or financial relationships that could be construed as a potential conflict of interest.

## Footnotes

1 Following the action editor’s suggestions and referring to the work of Becker et al. (2016), we have re-done the analyses without control variables of age, gender, education, and leader-follower dyadic tenure; the results remained substantially unchanged with regards to tests of hypotheses and the strength and direction of main relationships.

2 Within-construct item (for example, items corresponding to the strategic optimism scale with other items pertaining to the same scale) residuals were allowed to correlate. Without those modification indices, the results of the model fit were chi-square (176) = 361,788, CFI = 0.918, SRMR = 0.0756, and RMSEA = 0.060.

## References

[B1] Aiken L. S. West S. G. Reno R. R. (1991). *Multiple Regression: Testing and Interpreting Interactions.* Newbury Park, CA: Sage.

[B2] Albrecht S. L. Andreetta M. (2011). The influence of empowering leadership, empowerment and engagement on affective commitment and turnover intentions in community health service workers: test of a model. *Leadersh. Health Serv.* 24 228–237. 10.1108/17511871111151126

[B3] Avery D. R. McKay P. F. Wilson D. C. (2007). Engaging the aging workforce: the relationship between perceived age similarity, satisfaction with coworkers, and employee engagement. *J. Appl. Psychol.* 92 1542–1556. 10.1037/0021-9010.92.6.1542 18020795

[B4] Avey J. B. Avolio B. J. Luthans F. (2011). Experimentally analyzing the impact of leader positivity on follower positivity and performance. *Leadersh. Q.* 22 282–294. 10.1016/j.leaqua.2011.02.004

[B5] Avey J. B. Luthans F. Jensen S. M. (2009). Psychological capital: a positive resource for combating employee stress and turnover. *Hum. Resour. Manag.* 48 677–693. 10.1002/hrm.20294

[B6] Baer R. A. Smith G. T. Hopkins J. Krietemeyer J. Toney L. (2006). Using self-report assessment methods to explore facets of mindfulness. *Assessment* 13 27–45. 10.1177/1073191105283504 16443717

[B7] Becker T. E. Atinc G. Breaugh J. A. Carlson K. D. Edwards J. R. Spector P. E. (2016). Statistical control in correlational studies: 10 essential recommendations for organizational researchers. *J. Organ. Behav.* 37 157–167. 10.1002/job.2053

[B8] Biddle B. J. (1979). *Role Theory: Expectations, Identities, and Behaviors.* New York, NY: Academic Press.

[B9] Bishop S. R. Lau M. Shapiro S. Carlson L. Anderson N. D. Carmody J. (2004). Mindfulness: a proposed operational definition. *Clin. Psychol.* 11 230–241. 10.1093/clipsy.bph077

[B10] Blaskovics B. (2014). Impact of leadership styles on project success – the case of a multinational company. *Dyn. Relationsh. Manag. J.* 3 21–36. 10.17708/DRMJ.2014.v03n02a02

[B11] Bligh M. C. (2011). “Followership and follower-centered approaches,” in *The Sage Handbook of Leadership*, eds Bryman A. Collinson D. Grint K. Jackson B. Uhl-Bien M. (London: SAGE Publications), 393–403.

[B12] Brouer R. L. Duke A. Treadway D. C. Ferris G. R. (2009). The moderating effect of political skill on the demographic dissimilarity: leader-member exchange quality relationship. *Leadersh. Q.* 20 61–69. 10.1016/j.leaqua.2009.01.015

[B13] Brown K. W. Ryan R. M. (2003). The benefits of being present: mindfulness and its role in psychological well-being. *J. Pers. Soc. Psychol.* 84 822–848. 10.1037/0022-3514.84.4.822 12703651

[B14] Bunjak A. Černe M. (2018). The role of leader-follower defensive pessimism (in)congruence in fostering perceptions of followers’ isolation. *Econ. Bus. Rev.* 20 129–157.

[B15] Buunk B. P. Doosje B. J. Jans L. G. Hopstaken L. E. (1993). Perceived reciprocity, social support, and stress at work: the role of exchange and communal orientation. *J. Pers. Soc. Psychol.* 65 801–811. 10.1037/0022-3514.65.4.801

[B16] Cable D. M. Edwards J. R. (2004). Complementary and supplementary fit: a theoretical and empirical integration. *J. Appl. Psychol.* 89 822–834. 10.1037/0021-9010.89.5.822 15506863

[B17] Cameron K. McNaughtan J. (2014). Positive organizational change. *J. Appl. Behav. Sci.* 50 445–462. 10.1177/0021886314549922

[B18] Carlson E. N. (2013). Overcoming the barriers to self-knowledge: mindfulness as a path to seeing yourself as you really are. *Perspect. Psychol. Sci.* 8 173–186. 10.1177/1745691612462584 26172498

[B19] Cavazotte F. Moreno V. Hickmann M. (2012). Effects of leader intelligence, personality and emotional intelligence on transformational leadership and managerial performance. *Leadersh. Q.* 23 443–455. 10.1016/j.leaqua.2011.10.003

[B20] Černe M. Dimovski V. Marič M. Penger S. Škerlavaj M. (2014). Congruence of leader self-perceptions and follower perceptions of authentic leadership: understanding what authentic leadership is and how it enhances employees’ job satisfaction. *Aust. J. Manag.* 39 453–471. 10.1177/0312896213503665

[B21] Chalofsky N. Krishna V. (2009). Meaningfulness, commitment, and engagement: the intersection of a deeper level of intrinsic motivation. *Adv. Dev. Hum. Resour.* 11 189–203. 10.1177/1523422309333147

[B22] Chang E. C. Chang R. Sanna L. J. (2009). Optimism, pessimism, and motivation: relations to adjustment. *Soc. Pers. Psychol. Compass* 3 494–506. 10.1111/j.1751-9004.2009.00190.x

[B23] Cheek N. N. Norem J. K. (2017). Holistic thinkers anchor less: exploring the roles of self-construal and thinking styles in anchoring susceptibility. *Pers. Individ. Differ.* 115 174–176. 10.1016/j.paid.2016.01.034

[B24] Chiesa A. (2013). The difficulty of defining mindfulness: current thought and critical issues. *Mindfulness* 4 255–268. 10.1007/s12671-012-0123-4

[B25] Cornelis I. Van Hiel A. De Cremer D. (2006). Effects of procedural fairness and leader support on interpersonal relationships among group members. *Group Dyn. Theory Res. Pract.* 10 309–328. 10.1037/1089-2699.10.4.309

[B26] Costantini A. De Paola F. Ceschi A. Sartori R. Meneghini A. M. Di Fabio A. (2017). Work engagement and psychological capital in the Italian public administration: a new resource-based intervention programme. *SA J. Ind. Psychol.* 43 1–11. 10.4102/sajip.v43i0.1413

[B27] Dekeyser M. Raes F. Leijssen M. Leysen S. Dewulf D. (2008). Mindfulness skills and interpersonal behaviour. *Pers. Individ. Differ.* 44 1235–1245. 10.1016/j.paid.2007.11.018

[B28] Dinh J. E. Lord R. G. (2012). Implications of dispositional and process views of traits for individual difference research in leadership. *Leadersh. Q.* 23 651–669. 10.1016/j.leaqua.2012.03.003

[B29] Dutton J. E. Ragins B. R. (eds). (2017). “Positive relationships at work: an introduction and invitation,” in *Exploring Positive Relationships at Work*, (New York, NY: Psychology Press), 2–24. 10.4324/9781315094199

[B30] Dvir T. Shamir B. (2003). Follower developmental characteristics as predicting transformational leadership: a longitudinal field study. *Leadersh. Q.* 14 327–344. 10.1016/S1048-9843(03)00018-3

[B31] Eagly A. H. Karau S. J. (2002). Role congruity theory of prejudice toward female leaders. *Psychol. Rev.* 109 573–598. 10.1037/0033-295X.109.3.573 12088246

[B32] Eberth J. Sedlmeier P. (2012). The effects of mindfulness meditation: a meta-analysis. *Mindfulness* 3 174–189. 10.1007/s12671-012-0101-x

[B33] Edwards D. J. McEnteggart C. Barnes-Holmes Y. Lowe R. Evans N. Vilardaga R. (2017). The impact of mindfulness and perspective-taking on implicit associations toward the elderly: a relational frame theory account. *Mindfulness* 8 1615–1622. 10.1007/s12671-017-0734-x 29399210 PMC5796557

[B34] Edwards J. R. (2002). “Alternatives to difference scores: polynomial regression and response surface methodology,” in *The Jossey-Bass Business & Management Series. Measuring and Analyzing Behavior in Organizations: Advances in Measurement and Data Analysis*, eds Drasgow F. Schmitt N. (San Francisco, CA: Jossey-Bass), 350–400.

[B35] Edwards J. R. Cable D. M. (2009). The value of value congruence. *J. Appl. Psychol.* 94 654–677. 10.1037/a0014891 19450005

[B36] Edwards J. R. Parry M. E. (1993). On the use of polynomial regression equations as an alternative to difference scores in organizational research. *Acad. Manag. J.* 36 1577–1613.

[B37] Engle E. M. Lord R. G. (1997). Implicit theories, self-schemas, and leader-member exchange. *Acad. Manag. J.* 40 988–1010.

[B38] Epitropaki O. Martin R. (2004). Implicit leadership theories in applied settings: factor structure, generalizability, and stability over time. *J. Appl. Psychol.* 89 293–310. 10.1037/0021-9010.89.2.293 15065976

[B39] Epitropaki O. Sy T. Martin R. Tram-Quon S. Topakas A. (2013). Implicit leadership and followership theories “in the wild”: taking stock of information-processing approaches to leadership and followership in organizational settings. *Leadersh. Q.* 24 858–881. 10.1016/j.leaqua.2013.10.005 30254592

[B40] Feldman G. Greeson J. Senville J. (2010). Differential effects of mindful breathing, progressive muscle relaxation, and loving-kindness meditation on decentering and negative reactions to repetitive thoughts. *Behav. Res. Ther.* 48 1002–1011. 10.1016/j.brat.2010.06.006 20633873 PMC2932656

[B41] Felfe J. Schyns B. (2010). Followers’ personality and the perception of transformational leadership: further evidence for the similarity hypothesis. *Br. J. Manag.* 21 393–410.

[B42] Fischer I. (2009). Friend or foe: subjective expected relative similarity as a determinant of cooperation. *J. Exp. Psychol.* 138 341–350. 10.1037/a0016073 19653794

[B43] Fleenor J. W. Smither J. W. Atwater L. E. Braddy P. W. Sturm R. E. (2010). Self–other rating agreement in leadership: a review. *Leadersh. Q.* 21 1005–1034. 10.1016/j.leaqua.2010.10.006

[B44] Funder D. C. Sneed C. D. (1993). Behavioral manifestations of personality: an ecological approach to judgmental accuracy. *J. Pers. Soc. Psychol.* 64 479–490. 10.1037/0022-3514.64.3.479 8468673

[B45] Galinsky A. D. Moskowitz G. B. (2000). Perspective-taking: decreasing stereotype expression, stereotype accessibility, and in-group favoritism. *J. Pers. Soc. Psychol.* 78 708–724. 10.1037/0022-3514.78.4.708 10794375

[B46] Gardner W. L. Avolio B. J. Luthans F. May D. R. Walumbwa F. (2005). “Can you see the real me?” A self-based model of authentic leader and follower development. *Leadersh. Q.* 16 343–372. 10.1016/j.leaqua.2005.03.003

[B47] Good D. J. Lyddy C. J. Glomb T. M. Bono J. E. Brown K. W. Duffy M. K. (2016). Contemplating mindfulness at work: an integrative review. *J. Manag.* 42 114–142. 10.1177/0149206315617003

[B48] Gooty J. Gavin M. Johnson P. D. Frazier M. L. Snow D. B. (2009). In the eyes of the beholder: transformational leadership, positive psychological capital, and performance. *J. Leadersh. Organ. Stud.* 15 353–367. 10.1177/1548051809332021

[B49] Gosling S. D. Rentfrow P. J. Swann W. B. Jr. (2003). A very brief measure of the Big-Five personality domains. *J. Res. Pers.* 37 504–528. 10.1177/1359105317720819 28810502 PMC5794667

[B50] Grabovac A. D. Lau M. A. Willett B. R. (2011). Mechanisms of mindfulness: a Buddhist psychological model. *Mindfulness* 2 154–166. 10.1007/s12671-011-0054-5

[B51] Graen G. B. Uhl-Bien M. (1995). Relationship-based approach to leadership: development of leader-member exchange (LMX) theory of leadership over 25 years: applying a multi-level multi-domain perspective. *Leadersh. Q.* 6 219–247. 10.1016/1048-9843(95)90036-5

[B52] Hall R. J. Lord R. G. (1995). Multi-level information-processing explanations of followers’ leadership perceptions. *Leadersh. Q.* 6 265–287. 10.1016/1048-9843(95)90010-1

[B53] Hanley A. W. Warner A. R. Dehili V. M. Canto A. I. Garland E. L. (2015). Washing dishes to wash the dishes: brief instruction in an informal mindfulness practice. *Mindfulness* 6 1095–1103. 10.1007/s12671-014-0360-9

[B54] Hansbrough T. K. Lord R. G. Schyns B. (2015). Reconsidering the accuracy of follower leadership ratings. *Leadersh. Q.* 26 220–237. 10.1016/j.leaqua.2014.11.006

[B55] Harrison D. A. Price K. H. Gavin J. H. Florey A. T. (2002). Time, teams, and task performance: changing effects of surface- and deep-level diversity on group functioning. *Acad. Manag. J.* 45 1029–1045. 10.5465/3069328

[B56] Hayes A. F. (2013). *Introduction to Mediation, Moderation, and Conditional Process Analysis: A Regression-Based Approach.* New York, NY: Guilford Press.

[B57] Howell J. M. Shamir B. (2005). The role of followers in the charismatic leadership process: relationships and their consequences. *Acad. Manag. Rev.* 30 96–112. 10.5465/amr.2005.15281435

[B58] Ilies R. Morgeson F. P. Nahrgang J. D. (2005). Authentic leadership and eudaemonic well-being: understanding leader–follower outcomes. *Leadersh. Q.* 16 373–394. 10.1016/j.leaqua.2005.03.002

[B59] Jha A. P. Krompinger J. Baime M. J. (2007). Mindfulness training modifies subsystems of attention. *Cogn. Affect. Behav. Neurosci.* 7 109–119. 10.3758/CABN.7.2.109 17672382

[B60] Jones J. R. Harter J. K. (2005). Race effects on the employee engagement-turnover intention relationship. *J. Leadersh. Organ. Stud.* 11 78–88. 10.1111/jocn.14311 29446550

[B61] Junker N. M. van Dick R. (2014). Implicit theories in organizational settings: a systematic review and research agenda of implicit leadership and followership theories. *Leadersh. Q.* 25 1154–1173. 10.1016/j.leaqua.2014.09.002

[B62] Kabat-Zinn J. (2003). Mindfulness-based interventions in context: past, present, and future. *Clin. Psychol.* 10 144–156. 10.1093/clipsy.bpg016

[B63] Keller T. (1999). Images of the familiar: individual differences and implicit leadership theories. *Leadersh. Q.* 10 589–607. 10.1016/S1048-9843(99)00033-8

[B64] Keller T. (2003). Parental images as a guide to leadership sensemaking: an attachment perspective on implicit leadership theories. *Leadersh. Q.* 14 141–160. 10.1016/S1048-9843(03)00007-9

[B65] Kristof-Brown A. L. Zimmerman R. D. Johnson E. C. (2005). Consequences of individuals’ fit at work: a meta-analysis of person-job, person-organization, person-group, and person-supervisor fit. *Pers. Psychol.* 58 281–342. 10.1111/j.1744-6570.2005.00672.x

[B66] Langer E. J. (2000). Mindful learning. *Curr. Dir. Psychol. Sci.* 9 220–223. 10.1111/1467-8721.00099

[B67] Langer E. J. Moldoveanu M. (2000). The construct of mindfulness. *J. Soc. Issues* 56 1–9. 10.1111/0022-4537.00148

[B68] Leroy H. Anseel F. Dimitrova N. G. Sels L. (2013). Mindfulness, authentic functioning, and work engagement: a growth modeling approach. *J. Vocat. Behav.* 82 238–247. 10.1016/j.jvb.2013.01.012

[B69] Leroy H. Anseel F. Gardner W. L. Sels L. (2015). Authentic leadership, authentic followership, basic need satisfaction, and work role performance: a cross-level study. *J. Manag.* 41 1677–1697. 10.1177/0149206312457822

[B70] Lindell M. K. Whitney D. J. (2001). Accounting for common method variance in cross-sectional research designs. *J. Appl. Psychol.* 86 114–121. 10.1037/0021-9010.86.1.114 11302223

[B71] Lord R. G. Brown D. J. (2001). Leadership, values, and subordinate self-concepts. *Leadersh. Q.* 12 133–152. 10.1007/s10943-011-9479-3 21424861

[B72] Lord R. G. Brown D. J. (2004). *Leadership Processes and Follower Self-Identity.* Mahwah, NJ: Erlbaum.

[B73] Lord R. G. Brown D. J. Harvey J. L. Hall R. J. (2001). Contextual constraints on prototype generation and their multilevel consequences for leadership perceptions. *Leadersh. Q.* 12 311–338. 10.1016/S1048-9843(01)00081-9

[B74] Lord R. G. Foti R. J. De Vader C. L. (1984). A test of leadership categorization theory: internal structure, information processing, and leadership perceptions. *Organ. Behav. Hum. Perform.* 34 343–378. 10.1016/0030-5073(84)90043-6

[B75] Lord R. G. Hall R. (2003). “Identity, leadership categorization, and leadership schema,” in *Leadership and Power: Identity Processes in Groups and Organizations*, eds Knippenberg D. van Hogg M. (London: Sage), 48–64.

[B76] Lord R. G. Maher K. J. (1991). “Cognitive theory in industrial and organizational psychology,” in *Handbook of Industrial and Organizational Psychology* Vol. 2 eds Dunnette M. D. Hough L. M. (Palo Alto, CA: Consulting Psychologists Press), 1–62.

[B77] Luthans F. Avey J. B. Avolio B. J. Peterson S. J. (2010). The development and resulting performance impact of positive psychological capital. *Hum. Resour. Dev. Q.* 21 41–67. 10.1002/hrdq.20034

[B78] Luthans F. Avolio B. J. (2009). The “point” of positive organizational behavior. *J. Organ. Behav.* 30 291–307. 10.1002/job.589

[B79] Luthans F. Avolio B. J. Avey J. B. Norman S. M. (2007). Positive psychological capital: measurement and relationship with performance and satisfaction. *Pers. Psychol.* 60 541–572. 10.1136/bmjqs-2017-006847 28971881 PMC5867435

[B80] Malinowski P. Lim H. J. (2015). Mindfulness at work: positive affect, hope, and optimism mediate the relationship between dispositional mindfulness, work engagement, and well-being. *Mindfulness* 6 1250–1262. 10.1007/s12671-015-0388-5

[B81] Mauno S. Kinnunen U. Mäkikangas A. Nätti J. (2005). Psychological consequences of fixed-term employment and perceived job insecurity among health care staff. *Eur. J. Work Organ. Psychol.* 14 209–237. 10.1080/13594320500146649

[B82] Medvedeff M. E. Lord R. G. Shamir B. Pallai R. Bligh M. C. Uhl-Bien M. (2007). “Implicit leadership theories as dynamic processing structures,” in *Followercentered Perspectives on Leadership: A Tribute to the Memory of James R. Meindl*, eds Shamir B. Pillai R. Bligh M. C. Uhl-Bien M. (Greenwich, CT: Information Age), 19–50.

[B83] Meuser J. D. Gardner W. L. Dinh J. E. Hu J. Liden R. C. Lord R. G. (2016). A network analysis of leadership theory: the infancy of integration. *J. Manag.* 42 1374–1403. 10.1177/0149206316647099

[B84] Mikulas W. L. (2011). Mindfulness: significant common confusions. *Mindfulness* 2 1–7. 10.1007/s12671-010-0036-z

[B85] Montoya R. M. Horton R. S. (2013). A meta-analytic investigation of the processes underlying the similarity-attraction effect. *J. Soc. Pers. Relat.* 30 64–94. 10.1177/0265407512452989

[B86] Montoya R. M. Horton R. S. Kirchner J. (2008). Is actual similarity necessary for attraction? A meta-analysis of actual and perceived similarity. *J. Soc. Pers. Relat.* 25 889–922. 10.1177/0265407508096700

[B87] Mrazek M. D. Franklin M. S. Phillips D. T. Baird B. Schooler J. W. (2013). Mindfulness training improves working memory capacity and GRE performance while reducing mind wandering. *Psychol. Sci.* 24 776–781. 10.1177/0956797612459659 23538911

[B88] Norem J. K. (2001). “Defensive pessimism, optimism, and pessimism,” in *Optimism and Pessimism: Implications for Theory, Research, and Practice*, ed. Chang E. C. (Washington, DC: American Psychological Association), 77–100. 10.1037/10385-004

[B89] Norem J. K. Illingworth K. S. (1993). Strategy-dependent effects of reflecting on self and tasks: some implications of optimism and defensive pessimism. *J. Pers. Soc. Psychol.* 65 822–835. 10.1037/0022-3514.65.4.822

[B90] Oakes P. J. Turner J. C. Haslam S. A. (1991). Perceiving people as group members: the role of fit in the salience of social categorizations. *Br. J. Soc. Psychol.* 30 125–144. 10.1111/j.2044-8309.1991.tb00930.x

[B91] Parker S. K. Axtell C. M. (2001). Seeing another viewpoint: antecedents and outcomes of employee perspective taking. *Acad. Manag. J.* 44 1085–1100.

[B92] Pelham B. W. Carvallo A. Jones J. T. (2005). Implicit egotism. *Curr. Dir. Psychol. Sci.* 14 106–110. 10.1111/j.0963-7214.2005.00344.x

[B93] Pelham B. W. Mirenberg M. C. Jones J. T. (2002). Why Susie sells seashells by the seashore: implicit egotism and major life decisions. *J. Pers. Soc. Psychol.* 82 469–487. 10.1037/0022-3514.82.4.469 11999918

[B94] Pierce J. R. Aguinis H. (2013). The too-much-of-a-good-thing effect in management. *J. Manag.* 39 313–338. 10.1037/pspp0000147 28557471

[B95] Pircher Verdorfer A. (2016). Examining mindfulness and its relations to humility, motivation to lead, and actual servant leadership behaviors. *Mindfulness* 7 950–961. 10.1007/s12671-016-0534-8

[B96] Pirola-Merlo A. Härtel C. Mann L. Hirst G. (2002). How leaders influence the impact of affective events on team climate and performance in R&D teams. *Leadersh. Q.* 13 561–581. 10.1016/S1048-9843(02)00144-3

[B97] Preacher K. J. Hayes A. F. (2004). SPSS and SAS procedures for estimating indirect effects in simple mediation models. *Behav. Res. Methods Instr. Comput.* 36 717–731. 10.3758/BF03206553 15641418

[B98] Reis H. T. Collins W. A. Berscheid E. (2000). The relationship context of human behavior and development. *Psychol. Bull.* 126 844–872. 10.1037/0033-2909.126.6.844 11107879

[B99] Rich B. L. Lepine J. A. Crawford E. R. (2010). Job engagement: antecedents and effects on job performance. *Acad. Manag. J.* 53 617–635. 10.5465/amj.2010.51468988

[B100] Ritter B. A. Lord R. G. (2007). The impact of previous leaders on the evaluation of new leaders: an alternative to prototype matching. *J. Appl. Psychol.* 92 1683–1695. 10.1037/0021-9010.92.6.1683 18020805

[B101] Rowold J. Schlotz W. (2009). Transformational and transactional leadership and followers’ chronic stress. *Leadersh. Rev.* 9 35–48.

[B102] Schaufeli W. Salanova M. (2007). “Work engagement: an emerging psychological concept and its implications for organizations,” in *Research in Social Issues in Management: Managing Social and Ethical Issues in Organizations* Vol. 5 eds Gilliland S. W. Steiner D. D. Skarlicki D. P. (Greenwich, CT: Information Age Publishers), 135–177.

[B103] Schaufeli W. B. Salanova M. González-Romá V. Bakker A. B. (2002). The measurement of engagement and burnout: a two sample confirmatory factor analytic approach. *J. Happiness Stud.* 3 71–92. 10.1023/A:1015630930326

[B104] Scheier M. E. Carver C. S. (1987). Dispositional optimism and physical well-being: the influence of generalized outcome expectancies on health. *J. Pers.* 55 169–210. 10.1111/j.1467-6494.1987.tb00434.x 3497256

[B105] Schyns B. Day D. (2010). Critique and review of leader–member exchange theory: issues of agreement, consensus, and excellence. *Eur. J. Work Organ. Psychol.* 19 1–29. 10.1080/13594320903024922

[B106] Schyns B. Meindl J. R. (eds). (2005). “An overview of implicit leadership theories and their application in organization practice,” in *Implicit Leadership Theories: Essays and Explorations*, (Greenwich, CT: Information Age Publishing), 15–36.

[B107] Schyns B. Sanders K. (2007). In the eyes of the beholder: personality and the perception of leadership 1. *J. Appl. Soc. Psychol.* 37 2345–2363. 10.1037/pag0000186 28891667

[B108] Scott K. A. Brown D. J. (2006). Female first, leader second? Gender bias in the encoding of leadership behavior. *Organ. Behav. Hum. Decis. Process.* 101 230–242. 10.1016/j.obhdp.2006.06.002

[B109] Seligman M. E. Steen T. A. Park N. Peterson C. (2005). Positive psychology progress: empirical validation of interventions. *Am. Psychol.* 60 410–421. 10.1037/0003-066X.60.5.410 16045394

[B110] Seligman M. E. P. (2011). *Learned Optimism: How to Change Your Mind and Your Life.* New York, NY: Vintage.

[B111] Shamir B. (2007). “From passive recipients to active co-producers: the roles of followers in the leadership process,” in *Follower-Centered Perspectives on Leadership: A Tribute to J. R. Meindl*, eds Shamir B. Pillai R. Bligh M. Uhl-Bien M. (Stamford, CT: Information Age Publishing).

[B112] Shondrick S. J. Dinh J. E. Lord R. G. (2010). Developments in implicit leadership theory and cognitive science: applications to improving measurement and understanding alternatives to hierarchical leadership. *Leadersh. Q.* 21 959–978. 10.1016/j.leaqua.2010.10.004

[B113] Sluss D. M. Ashforth B. E. (2007). Relational identity and identification: defining ourselves through work relationships. *Acad. Manag. Rev.* 32 9–32. 10.5465/amr.2007.23463672

[B114] Spencer S. M. Norem J. K. (1996). Reflection and distraction defensive pessimism, strategic optimism, and performance. *Pers. Soc. Psychol. Bull.* 22 354–365. 10.1177/0146167296224003

[B115] Sy T. (2010). What do you think of followers? Examining the content, structure, and consequences of implicit followership theories. *Organ. Behav. Hum. Decis. Process.* 113 73–84. 10.1016/j.obhdp.2010.06.001

[B116] Taylor S. E. Brown J. D. (1988). Illusion and well-being: a social psychological perspective on mental health. *Psychol. Bull.* 103 193–210. 10.1037/0033-2909.103.2.193 3283814

[B117] Teasdale J. D. Moore R. G. Hayhurst H. Pope M. Williams S. Segal Z. V. (2002). Metacognitive awareness and prevention of relapse in depression: empirical evidence. *J. Consult. Clin. Psychol.* 70 275–287. 10.1037/0022-006X.70.2.275 11952186

[B118] Tims M. Bakker A. B. Xanthopoulou D. (2011). Do transformational leaders enhance their followers’ daily work engagement? *Leadersh. Q.* 22 121–131. 10.1016/j.leaqua.2010.12.011

[B119] Toma C. Corneille O. Yzerbyt V. (2012). Holding a mirror up to the self: egocentric similarity beliefs underlie social projection in cooperation. *Pers. Soc. Psychol. Bull.* 38 1259–1271. 10.1177/0146167212449022 22700241

[B120] Uhl-Bien M. Riggio R. E. Lowe K. B. Carsten M. K. (2014). Followership theory: a review and research agenda. *Leadersh. Q.* 25 83–104. 10.1016/j.leaqua.2013.11.007

[B121] Van Breukelen W. Konst D. Van Der Vlist R. (2002). Effects of LMX and differential treatment on work unit commitment. *Psychol. Rep.* 91 220–230. 10.2466/PR0.91.5.220-230 12353784

[B122] van Gils S. van Quaquebeke N. van Knippenberg D. (2010). The X-factor: on the relevance of implicit leadership and followership theories for leader–member exchange agreement. *Eur. J. Work Organ. Psychol.* 19 333–363. 10.1080/13594320902978458

[B123] van Quaquebeke N. van Knippenberg D. Eckloff T. (2011). Individual differences in the leader categorization to openness to influence relationship: the role of followers’ self-perception and social comparison orientation. *Group Process. Intergroup Relat.* 14 605–622. 10.1177/1368430210391311

[B124] Von Hippel W. Trivers R. (2011). Reflections on self-deception. *Behav. Brain Sci.* 34 41–56. 10.1348/147608309X450508 21288379

[B125] Wachs K. Cordova J. V. (2007). Mindful relating: exploring mindfulness and emotion repertoires in intimate relationships. *J. Marital Fam. Ther.* 33 464–481. 10.1111/j.1752-0606.2007.00032.x 17935530

[B126] Watson D. Hubbard B. Wiese D. (2000). Self–other agreement in personality and affectivity: the role of acquaintanceship, trait visibility, and assumed similarity. *J. Pers. Soc. Psychol.* 78 546–558. 10.1037/0022-3514.78.3.546 10743880

[B127] Youssef C. M. Luthans F. (2007). Positive organizational behavior in the workplace: the impact of hope, optimism, and resilience. *J. Manag.* 33 774–800. 10.1177/0149206307305562

[B128] Zeidan F. Johnson S. K. Diamond B. J. David Z. Goolkasian P. (2010). Mindfulness meditation improves cognition: evidence of brief mental training. *Conscious. Cogn.* 19 597–605. 10.1016/j.concog.2010.03.014 20363650

